# Effects of Cu/Er on Tensile Properties of Cast Al-Si Alloy at Low Temperature

**DOI:** 10.3390/ma16030902

**Published:** 2023-01-17

**Authors:** Huidi Zhang, Bin Chen, Jianfei Hao, Huishu Wu, Ming Chen, Weirong Li, Runxia Li, Biao Wang

**Affiliations:** 1 Faculty of Materials Science and Engineering, Dongguan University of Technology, Dongguan 523808, China; 2 Faculty of Materials Science and Engineering, Shenyang University of Technology, Shenyang 110870, China; 3 Qingniao Technology Co., Ltd., Dongguan 523000, China; 4 Dongguan Eontec Co., Ltd., Dongguan 523000, China

**Keywords:** cast Al-Si alloy, alloy elements, low-temperature tensile properties, microstructure

## Abstract

The current protocol presents the effects of the addition of Cu, rare earth Er, and Cu-Er composite elements on the microstructure of the Al-10Si-0.3Mg alloy. The variations in their low-temperature tensile properties were also investigated. The addition of rare earth Er elements, Cu elements, and Cu-Er composite elements increased the strength of all three groups of alloys when stretched at low temperatures (−60 °C). Further, the elongation of the alloy increased with the addition of Er, while the elongation of the other two groups decreased. The low-temperature (−60 °C) tensile strength of the alloy with the same composition was higher than that at room temperature (20 °C), but the elongation decreased. Notably, by adding rare earth Er to the Al-10Si-0.3Mg alloy, the three-dimensional morphology was changed from coarse dendritic to fine fibrous, the secondary dendritic arm spacing (SDAS) of the alloy was reduced, and the grains were refined. The Al_2_Cu phase, Al-Si-Cu-Mg quaternary phase, and Cu-rich phase appeared in the alloy with the addition of Cu elements, but the Si phase morphology and α-Al dendrites were not significantly improved. Interestingly, the Si phase morphology of the alloy was improved by adding Cu-Er composite elements, and SDAS was reduced. Still, the Al_2_Cu phase, Al-Si-Cu-Mg quaternary phase, and Cu-rich phase were not much improved.

## 1. Introduction

Cast Al-Si alloys account for about 85% of the total cast aluminum-based alloy market usage. They have been widely used in manufacturing components for aerospace and high-speed trains [1,2,3]. With high-speed trains constantly penetrating northeastern China and going through Russia and other regions with freezing winters, the overall performance of Al-Si alloy materials used in high-speed trains is required to be higher at low temperatures [4,5,6]. Rincon et al. [7] discovered that the second phase in the A319 alloy, such as Mg_2_Si and Al_2_Cu, has a significant influence on the mechanical properties of Al-Si alloys at low temperatures. Gokhale et al. [8] demonstrated that the decrease in elongation of hypoeutectic Al-Si alloys is closely related to Si phase fracture under low-temperature conditions. Ma G. et al. [2] discovered that as the tensile temperature decreased from 20 °C to −60 °C, the strength of the A356 alloy increased while the ductility decreased. Therefore, optimizing the composition and analyzing the performance of Al-Si alloy materials in low-temperature environments is very necessary.

Cu is the most common element used in the composition optimization of aluminum alloys. The previous studies [9,10,11,12] demonstrated that adding a Cu element in Al-Si-Mg alloys formed the Mg_2_Si phase, θ (Al_2_Cu) phase, and Q phase in the alloys, which precipitated to different degrees during the aging process. At the low ratio of Cu/Mg of the alloy, the precipitation of the Mg_2_Si phase increased, and at the high ratio of Cu/Mg of the alloy, it favorably promoted the precipitation of θ and Q phases. To enhance the thermal resistance of the alloy, a higher content of Cu elements and a relatively low content of Mg elements were added. In the study reported by Stadler et al. [13], it was found that the strength of the Al-Si-Cu alloy increased with increasing Cu content in the range of 1–3 wt.%. The study concluded that the strengthening impact of the Al_2_Cu phase was substantial, but adding Cu elements reduced the ductility and corrosion resistance of the alloy.

Both rare earth elements Er and Sc are microalloying elements that primarily produce grain refinement and strengthening through their own precipitation phase formation [14]. Furthermore, the rare earth Er and rare earth Sc elements play similar roles in aluminum alloys. However, rare earth Er elements are generally less expensive than Sc elements [15]. Previous studies [16,17,18,19] showed that adding rare earth Er elements to aluminum alloys resulted in the formation of Al_3_Er, which has the same structure as the strengthening phase Al_3_Sc. Both are L1_2_ structural phases with good strengthening and excellent thermal stability and corrosion resistance. According to the findings, rare earth Er elements can significantly refine the microstructure of aluminum alloys, and nanoscale Al_3_Er particles can effectively inhibit dislocation movement, improving the alloy’s overall performance. Colombo et al. [20] discovered that adding 0.3 wt.% Er had a significant modification effect on the microstructure of the A356 alloy. In a similar study, Li J. et al. [21] demonstrated that by adding 0.1 wt.% Er, the Si grains of the A356 alloy were refined to a certain extent, and the optimum strength was achieved by adding 0.3 wt.% Er. Yang et al. [22] discovered that while the addition of rare earth Er could increase the recrystallization temperature of the Al-4Cu alloy, it could not improve the strength, and could even reduce it to some extent. The study of Er-containing aluminum alloys has recently received a lot of attention. Several studies have been conducted on the addition of rare earth Er elements to Al-Mg series alloys, Al-Zn-Mg series alloys, and Al-Cu series alloys. Nonetheless, there have been fewer studies on the addition of Er elements to Al-Si series alloys [15]. Thus, it is worthwhile to investigate the effects of rare earth elements Er and Cu on the tensile properties of cast Al-Si alloy at low temperatures.

In this paper, the composition of the Al-10Si-0.3Mg alloy is optimized by adding Cu, rare earth Er, and Cu-Er composite elements. Further, the impact of the addition of alloying elements on the microstructure of the alloy and the increase in the strength of the alloy at low temperatures (−60 °C) are briefly discussed. A reference for meeting the higher requirements of Al-Si alloy materials for use at low temperatures is also provided.

## 2. Materials and Methods

In the experimental method, the material is Al-10Si-0.3Mg alloy. Four groups of comparison experiments of alloys with different compositions were set up as follows: without adding alloying elements and with adding rare earth Er elements, Cu elements, and Cu-Er composite elements with different compositions. The chemical compositions and the grouping of the alloys are shown in Table 1. The alloy was melted in a resistance furnace. The pure Al and Al-30Si intermediate alloys were introduced into the crucible in the furnace, and the furnace temperature was controlled at 750 °C. The metal in the furnace was melted entirely and left to stand for some time. Further, the furnace temperature was lowered to 720 °C and then pressed into pure Mg and refined with C_2_Cl_6_ (0.8%) degassing. Later, Al-10Zr (0.3%) and Al-10Sr (0.3%) intermediate alloys were added to the metal liquid for modification and refinement. When the metal in the furnace was completely melted, it was gently stirred. After 10 min, the melt was poured into the metal mold, preheated to 200~300 °C, and allowed to cool and solidify naturally. Al-20Er (0.3%) intermediate alloy, Al-50Cu (3%) intermediate alloy, and a mixture of Al-20Er (0.3%) and Al-50Cu (3%) intermediate alloy were added during the above melting process, respectively.

The tensile sample was prepared by following the GB/T 13239–2006 standard. The tensile test at room temperature (20 °C) was carried out on a WGW-100H-type tensile machine. The tensile test was carried out at a low temperature (−60 °C) on the tensile test machine equipped with a temperature touch box. Tensile speeds were both 0.5 mm/min. During low-temperature stretching, liquid nitrogen was first introduced into the tensile test machine’s low-temperature holding tank. On the temperature touch display, the stretching temperature was set to −60 °C. Tensile specimens were placed in the holding tank for 10 min after the temperature was reduced to −60 °C and then subjected to low-temperature stretching.

To observe the Si phase stereo morphology, a 10% HF solution was prepared for deep corrosion of the alloy. The microstructure of the alloy was examined using an OLYMPUS GX51 inverted optical microscope (OM). S3400N and SU8010 scanning electron microscopes (SEM) were used to examine the second phase particles of the alloy and the morphology near the tensile fracture, and the secondary electron (SE) mode was used in SEM operations. The element distribution and the phase composition were analyzed using an energy-dispersive X-ray spectroscopy (EDS) instrument. The specimens were also analyzed with an XRD-7000 X-ray diffraction analyzer, which has a scanning range of 20°~90° and a scanning speed of 2°/min. Secondary dendrite arm spacing (SDAS) and porosity were measured using Image J software 1.53t.

## 3. Results and Discussion

### 3.1. Tensile Properties and Fracture Morphology

**Figure 1:**
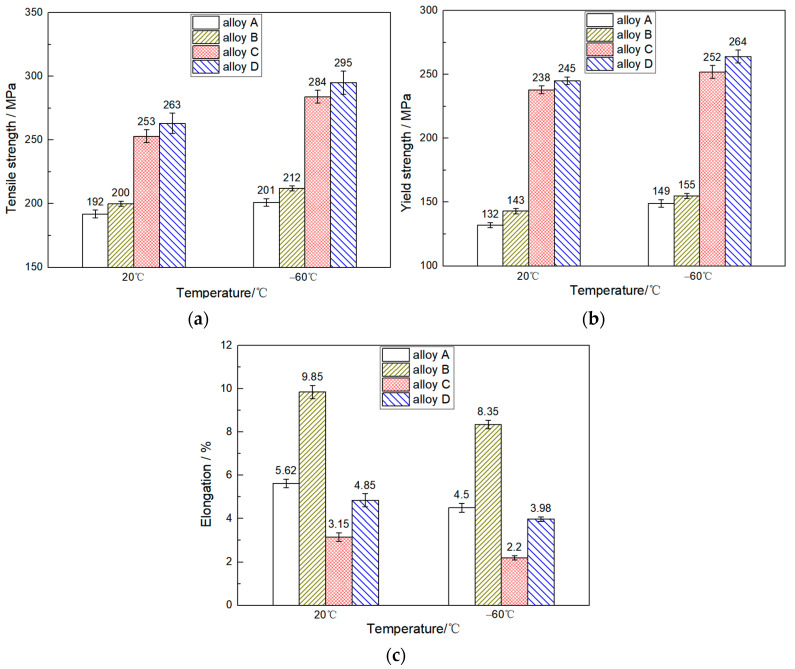
Effect of alloying elements on the tensile properties of Al-Si alloys at room (20 °C) and low (−60 °C) temperature: (**a**) Tensile strength; (**b**) Yield strength; (**c**) Elongation.

Figure 1 shows the room temperature (20 °C) and low-temperature (−60 °C) tensile properties of the Al-10Si-0.3Mg alloy with the addition of rare earth Er elements, Cu elements, and Cu-Er composite elements. The result reveals that the strength of alloys A, B, C, and D was higher than 20 °C when stretched at −60 °C, while the elongation was the opposite. At a tensile temperature of −60 °C, the tensile strengths of alloys B, C, and D, with the addition of rare earth Er elements, Cu elements, and Cu-Er composite elements, reached 212 MPa, 284 MPa, and 295 MPa, respectively. Compared with the base alloy A without alloying elements, the increase was 5.47%, 41.29%, and 46.77%, respectively. The yield strength reached 155 MPa, 252 MPa, and 264 MPa, and increased by 4.03%, 69.13%, and 77.18%, respectively. The elongation of alloy B increased to 8.35%, which showed an 85.56% improvement compared to base alloy A. The elongation of alloys C and D decreased to 2.2% and 3.98%, respectively, representing a decrease of 51.11% and 11.56%. At a tensile temperature of 20 °C, the effect of alloying elements on the tensile properties of the Al-10Si-0.3Mg alloy was consistent with −60 °C. In addition, the porosities of alloys A, B, C, and D were all less than 0.5%, as determined by Image J software. 

**Figure 2:**
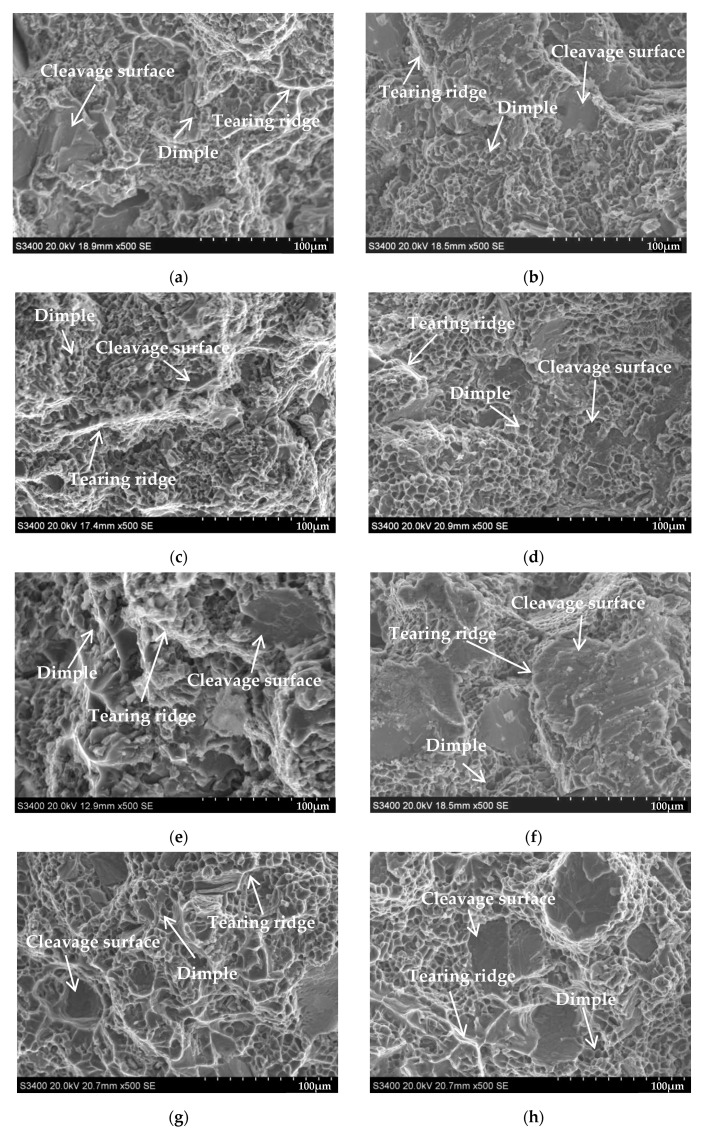
Effect of alloying elements on tensile fracture surface morphology of Al-Si alloys at room (20 °C) and low (−60 °C) temperature: (**a**) Al-10Si-0.3Mg, 20 °C; (**b**) Al-10Si-0.3Mg, −60 °C; (**c**) Al-10Si-0.3Mg-0.2Er, 20 °C; (**d**) Al-10Si-0.3Mg-0.2Er, −60 °C; (**e**) Al-10Si-3Cu-0.3Mg, 20 °C; (**f**) Al-10Si-3Cu-0.3Mg, −60 °C; (**g**) Al-10Si-3Cu-0.3Mg-0.2Er, 20 °C; (**h**) Al-10Si-3Cu-0.3Mg-0.2Er, −60 °C.

Figure 2 illustrates the fracture surface morphology of the Al-10Si-0.3Mg alloy with the addition of rare earth Er elements, Cu elements, and Cu-Er composite elements stretched at room temperature (20 °C) and low temperature (−60 °C). As seen in Figure 2a, the fracture of the base alloy A consists of many dimples, tearing ridges, and fewer cleavage surfaces at a tensile temperature of 20 °C. Compared with the base alloy A without the alloying elements, the number of dimples in the fracture of the alloy after the addition of rare earth Er elements significantly increased. In contrast, the number of cleavage surfaces substantially decreased, and the alloy exhibited ductility fracture characteristics, showing the ductility enhancement of the alloy (see Figure 2c). As seen in Figure 2e, adding Cu elements reduced the number of dimples in the fracture of the alloy. The number of tearing ridges was less, and the number of cleavage surfaces significantly increased, representing that the strength of the alloy increased while ductility decreased. Figure 2g reveals that adding Cu-Er composite elements slightly reduced the number of dimples in the alloy fracture. At the same time, the number of tearing ridges and cleavage surfaces increased.

The number of cleavage surfaces in the tensile fracture at −60 °C environment increased for the same alloy composition. In contrast, the number of tearing ridges and dimples decreased. Moreover, the effect of the addition of alloying elements on the dimples, tearing ridges, and cleavage surfaces in the fracture of the Al-Si-Mg alloy stretched at −60 °C was consistent with the stretching pattern at 20 °C (see Figure 2b,d,f,h).

### 3.2. Microstructure of the Alloys

**Figure 3:**
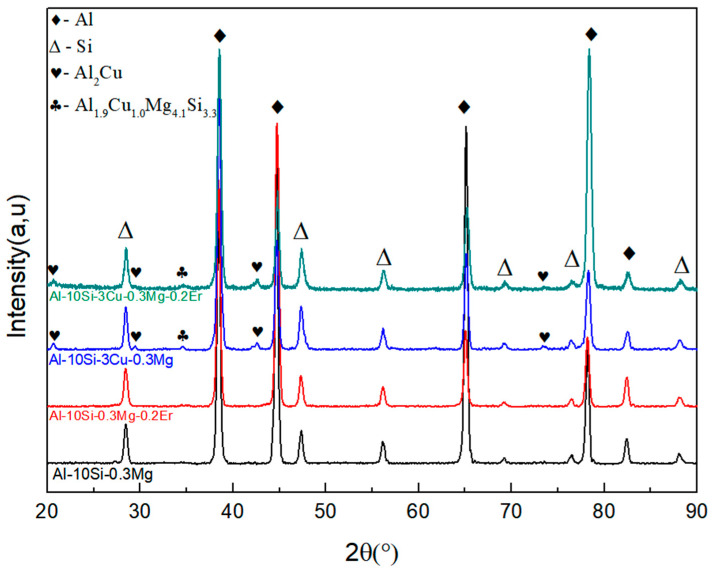
XRD patterns of Al-Si alloys with different compositions.

Figure 3 shows the XRD pattern of the alloys after adding rare earth Er elements, Cu elements, and Cu-Er composite elements to the Al-10Si-0.3Mg alloy, respectively. As shown in Figure 3, the Al-10Si-0.3Mg alloy consists mainly of the α-Al matrix and eutectic Si phase. After adding a small amount of Er elements to the alloy, the phase composition of the alloy did not change much. Notably, after adding the Cu elements, in addition to the original α-Al matrix and eutectic Si phase, the Al_2_Cu phase and Al_1.9_Cu_1.0_Mg_4.1_Si_3.3_ quaternary phase appeared in the alloy. After adding Cu-Er composite elements, the α-Al matrix, eutectic Si phase, Al_2_Cu phase, and Al_1.9_Cu_1.0_Mg_4.1_Si_3.3_ quaternary phase were also present in the alloy, but an Er-containing second phase was not found.

**Figure 4:**
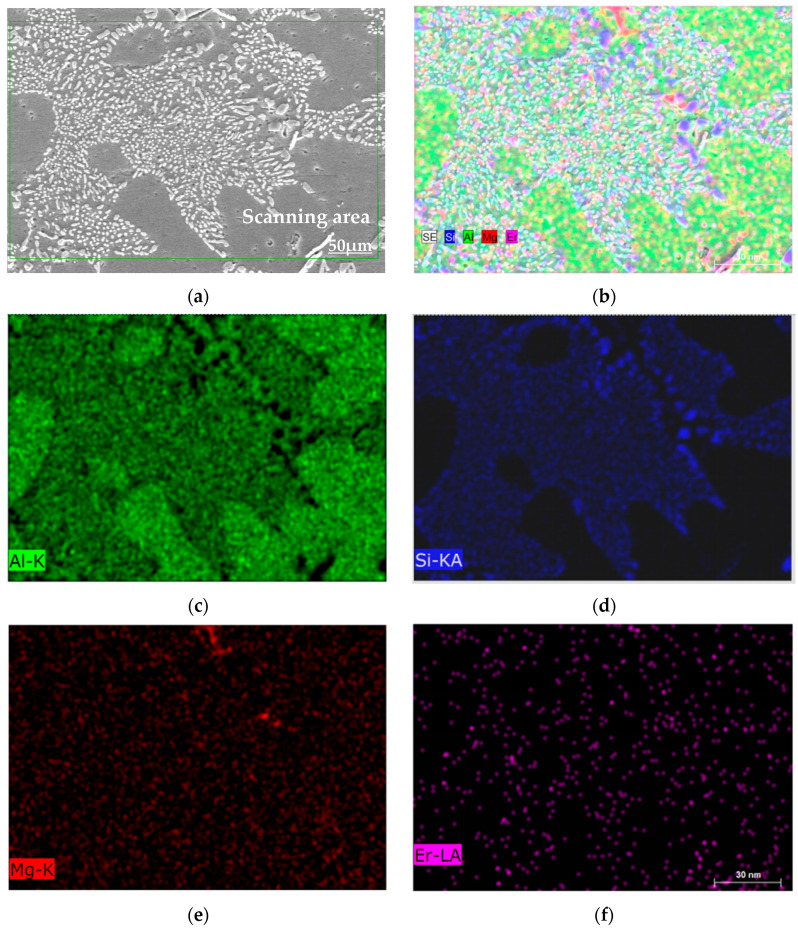
Element distribution analysis of Al-10Si-0.3Mg-0.2Er alloy through EDS: (**a**) Microstructure; (**b**) Overall distribution of Al, Si, Mg, and Er; (**c**) Distribution of Al; (**d**) Distribution of Si; (**e**) Distribution of Mg; (**f**) Distribution of Er.

The elemental distribution of the Al-10Si-0.3Mg-0.2Er alloy is depicted in Figure 4. The Al elements are abundantly distributed in the alloy as matrix elements, as shown in Figure 4c. Figure 4d depicts the segregation of Si elements. Both Mg and Er are dispersedly distributed in the α-Al matrix, shown in Figure 4e,f, which is consistent with the fact that no Mg-containing second phase and no Er-containing second phase were detected in the XRD pattern of the Al-10Si-0.3Mg-0.2Er alloy (Figure 3).

**Figure 5:**
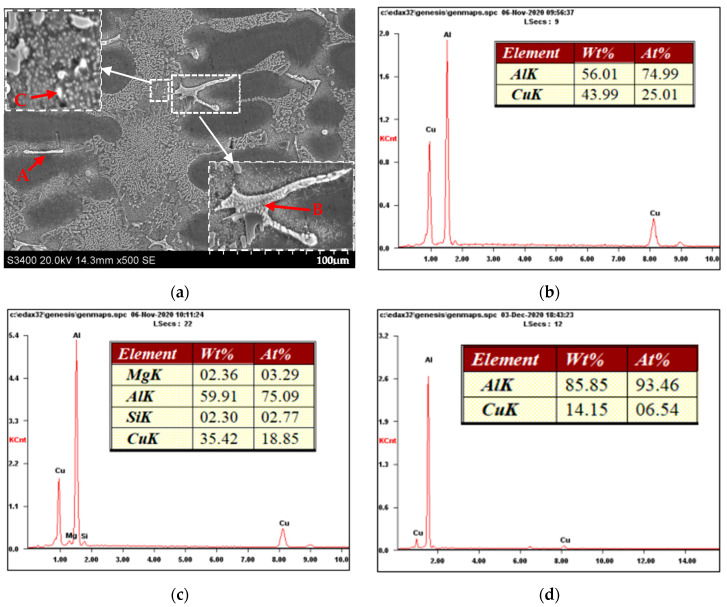
Microstructure of Al-10Si-3Cu-0.3Mg alloy and EDS energy spectrum analysis of its corresponding phases: (**a**) SEM microstructure of Al-10Si-3Cu-0.3Mg; (**b**) EDS spectrum corresponding to A in (**a**); (**c**) EDS spectrum corresponding to B in (**a**); (**d**) EDS spectrum corresponding to C in (**a**).

The microstructure and EDS energy spectrum analysis of the Al-10Si-3Cu-0.3Mg alloy are shown in Figure 5. By combining the results of Figure 5a,b with the XRD (Figure 3) results, it is discovered that the long striped phase A is the Al_2_Cu phase. The irregular net-like phase B is the Al-Si-Cu-Mg quaternary phase and the Al_2_Cu phase, as shown in Figure 5a,c, and combined with the XRD (Figure 3) results. Figure 5a,d, combined with the XRD (Figure 3), show a tiny granular phase C, which is a Cu-rich phase.

**Figure 6:**
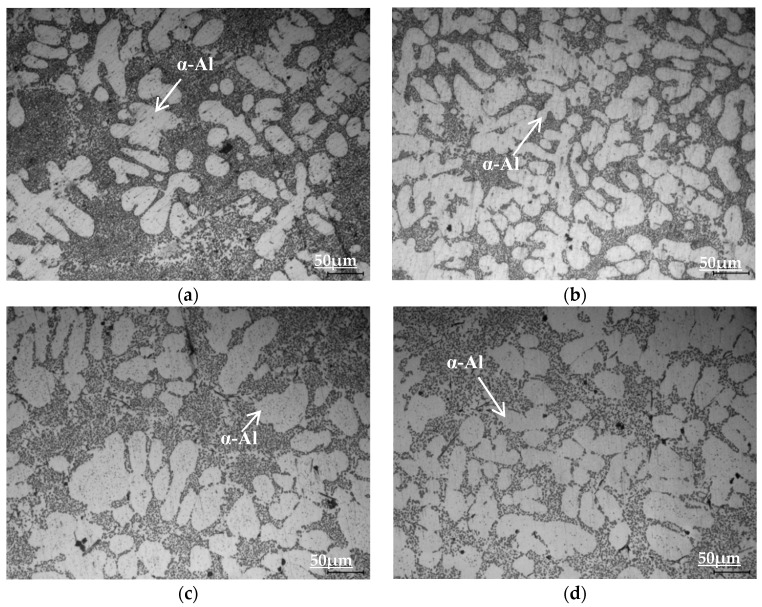
Effect of alloying elements on the OM microstructure of Al-Si alloys: (**a**) Al-10Si-0.3Mg; (**b**) Al-10Si-0.3Mg-0.2Er; (**c**) Al-10Si-3Cu-0.3Mg; (**d**) Al-10Si-3Cu-0.3Mg-0.2Er.

Figure 6 depicts the OM images of the Al-10Si-0.3Mg alloy after the addition of rare earth Er, Cu, and Cu-Er composite elements. Table 2 shows the results of the secondary dendrite arm spacing (SDAS) analysis. The addition of Er refines the coarse α-Al dendrites in the alloy (see Figure 6b) compared to the Al-10Si-0.3Mg alloy (see Figure 6a), and the secondary dendrite arm spacing (SDAS) is significantly reduced (see Table 2). The addition of Cu has no significant effect on the α-Al dendrites of the alloy (see Figure 6c), and the change in the SDAS is insignificant (see Table 2). The dendrites in the alloy are slightly refined after the addition of Cu-Er composite elements (see Figure 6d), and the SDAS is reduced when compared to the Al-10Si-3Cu-0.3Mg alloy (see Table 2). On the one hand, Er is enriched near the solid-liquid interface, inhibiting grain growth during alloy solidification [23]. The combination of Er and Al, on the other hand, can form a stable nanoscale Al_3_Er phase with an L1_2_-type crystal structure that can form a coherent relationship with the matrix. Al_3_Er was used as a heterogeneous nucleation core to increase the nucleation rate, lowering the SDAS and refining the alloy grains for fine grain strengthening [24,25].

**Figure 7:**
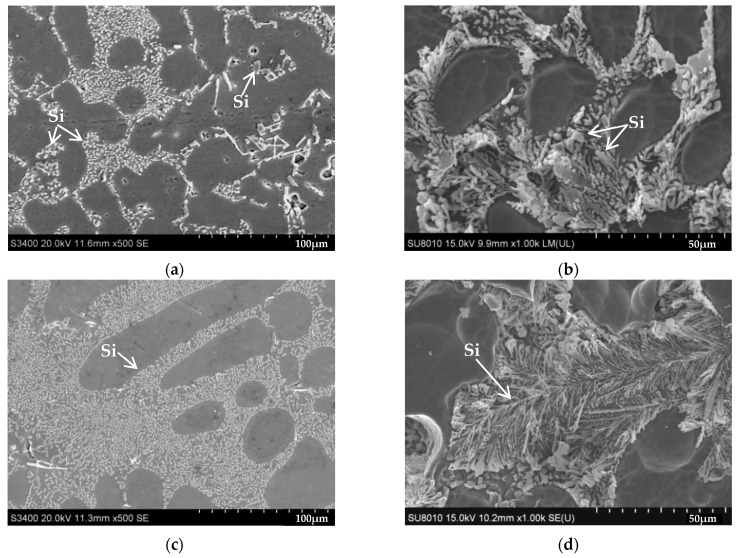

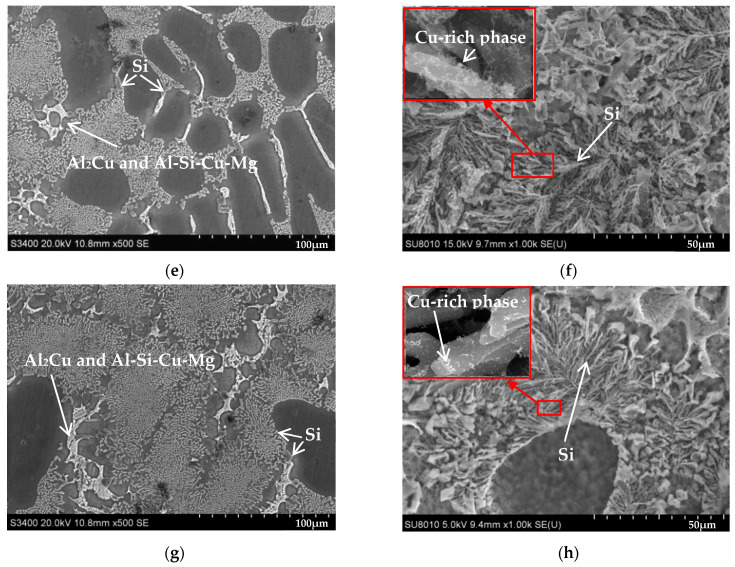
Effect of alloying elements on the SEM microstructure of Al-Si alloys: (**a**) Al-10Si-0.3Mg; (**b**) Al-10Si-0.3Mg (deep corrosion); (**c**) Al-10Si-0.3Mg-0.2Er; (**d**) Al-10Si-0.3Mg-0.2Er (deep corrosion); (**e**) Al-10Si-3Cu-0.3Mg; (**f**) Al-10Si-3Cu-0.3Mg (deep corrosion); (**g**) Al-10Si-3Cu-0.3Mg-0.2Er; (**h**) Al-10Si-3Cu-0.3Mg-0.2Er (deep corrosion).

Figure 7 illustrates the SEM microstructure of the Al-10Si-0.3Mg alloy after adding rare earth Er elements, Cu elements, and Cu-Er composite elements, respectively. Figure 7a,b show that the morphology of the Si phase in Al-10Si-0.3Mg alloy is irregular, where some appeared similar to small, short rods, and others looked similar to long, thick rods and plates. After deep corrosion, the Si phase seemed similar to a short rod and thick dendritic. Dislocations slipped and easily piled up near the coarse Si phase by subjecting the alloy to external force, resulting in stress concentration [26]. Moreover, the α-Al matrix was easily cut apart by the coarse Si phase, leading to poor mechanical properties of the alloy [27].

The Si phase in the alloy changed from the original coarse, long, rod-like, and plate-like shape to a fine, short, rod-like shape after Er elements were added (see Figure 7c). Figure 7d depicts the Si phase that appeared as fine fibrous after deep corrosion. It indicates that the Si phase morphology was improved by the addition of Er, which lowered the cut apart trend on the matrix, and reduced the local stress concentration in the alloy [28]. The tensile properties of the alloy were enhanced. During the alloy crystallization process, the Er atoms were enriched in the crystallization front of the Si phase and reduced the constitutional supercooling, inhibiting the rapid growth of the Si phase, thereby improving and refining the Si phase morphology [29]. A small amount of the atomic Er was also dissolved into the α-Al matrix, resulting in solid solution strengthening [30].

After adding Cu elements, the morphology of the Si phase in the alloy remained the same. Still, many coarse plate-like Si phases were observed, and on the Al_2_Cu phase and Al-Si-Cu-Mg quaternary phase a long, irregular, and net-like strip grew near the plate Si phase (see Figure 7e). The presence of the Al_2_Cu phase and Al-Si-Cu-Mg quaternary phase substantially increased the second phase strengthening effect, enhancing alloy strength. After deep corrosion, the Si phase was thick and dendritic, and it is worth noting the covering of the Si phase with a layer of a thin metallic phase (see Figure 7f). According to the EDS energy spectrum analysis from Figure 5a,d, it is thought that the thin metal particles should be a Cu-rich phase, which could fall off and attach to the dendrites of the Si phase during the deep corrosion process. These tiny Cu-rich phases strengthen the alloy matrix.

Figure 7g shows that adding Cu-Er composite elements slightly blunts the sharp edges of the Si phase in the alloy, and the Al_2_Cu phase and Al-Si-Cu-Mg quaternary phase were observed. Compared with the Al-10Si-3Cu-0.3Mg alloy, the morphology and size of the Al_2_Cu phase and Al-Si-Cu-Mg quaternary phase in Al-10Si-3Cu-0.3Mg-0.2Er alloy remained the same. Still, the morphology of the Si phase in the alloy was improved to a smaller and shorter rod shape. Figure 7h shows that the Si phase was also fine fibrous after deep corrosion, and the dendrites were covered with a thin metal phase.

### 3.3. Distribution of Dislocation Slip Zones in Alloy Fractures

**Figure 8:**
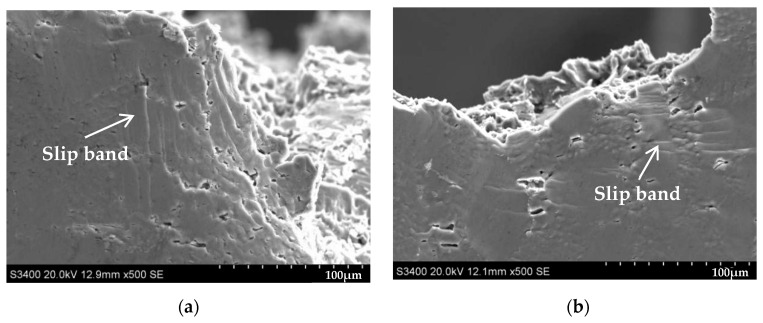

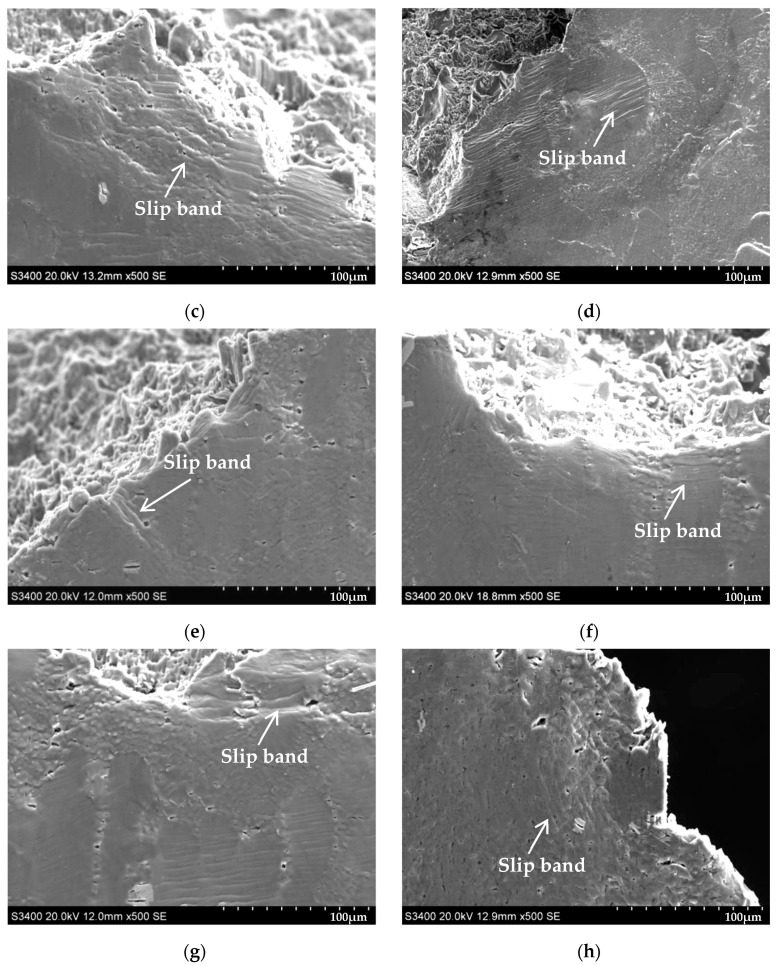
Effect of alloying elements on the slip bands near the tensile fracture of Al-Si alloys at room (20 °C) and low (−60 °C) temperature: (**a**) Al-10Si-0.3Mg, 20 °C; (**b**) Al-10Si-0.3Mg, −60 °C; (**c**) Al-10Si-0.3Mg-0.2Er, 20 °C; (**d**) Al-10Si-0.3Mg-0.2Er, −60 °C; (**e**) Al-10Si-3Cu-0.3Mg, 20 °C; (**f**) Al-10Si-3Cu-0.3Mg, −60 °C; (**g**) Al-10Si-3Cu-0.3Mg-0.2Er, 20 °C; (**h**) Al-10Si-3Cu-0.3Mg-0.2Er, −60 °C.

Figure 8 shows the distribution of slip bands near the tensile fracture at room temperature (20 °C) and low temperature (−60 °C) after the addition of rare earth Er elements, Cu elements, and Cu-Er composite elements to the Al-10Si-0.3Mg alloy, respectively. Figure 8a illustrates that the number of slip bands near the tensile fracture of the Al-10Si-0.3Mg alloy without alloying elements was relatively high at a tensile temperature of 20 °C, and the present form of slip bands was comparatively deep. As seen in Figure 8c, the number of slip bands near the tensile fracture of the alloy increased with the addition of rare earth Er elements, and the form of slip bands was further deepened and distributed in a step-like shape. Figure 8e shows that the number of slip bands near the tensile fracture of the alloy was considerably reduced, and the morphology turned out to be shallow with the addition of Cu elements. In comparison with the Al-10Si-3Cu-0.3Mg alloy, the number of slip bands near the tensile fracture of the alloy increased, and the morphology deepened after adding the Cu-Er composite elements (see Figure 8g).

The effect of adding alloying elements on the morphology and the number of slip bands near the fracture of the Al-10Si-0.3Mg alloy stretched at −60 °C was consistent with the stretching pattern at 20 °C. Furthermore, Figure 8b,d,f,h demonstrate that the number of slip bands near the tensile fracture was reduced, and the morphology became shallower at −60 °C compared to stretching at 20 °C for the same composition alloy.

The crystal deformation process resulted from dislocations sliding along a slip surface under applied tangential stress. As the resolved shear stress value in the slip system reached the maximum critical resolved shear stress, the slip system could be the first to slip. The movement of dislocations in the alloy would be subject to internal resistance, and the resistance *τ* to dislocations in the aluminum matrix is expressed as [31]:

(1) $$\tau =\tau_{p}+\tau_{\mu }$$

where *τ_p_* is the lattice resistance and *τ_µ_* is the resistance generated by the dislocation stress field. The magnitude of the lattice resistance *τ_p_* depends on the thermal vibrational state of the atom and is expressed as [32]:

(2) $$\tau_{p}=\tau_{0}\exp(-\frac{16\pi^{2}u^{2}r_{0}}{a^{3}})$$

where *τ*_0_ is the lattice resistance without considering thermal vibration; *u* is the thermal vibration amplitude of the atom; *a* is the distance between energy barriers; and *r*_0_ is the central radius of dislocation. As the temperature decreases, the thermal vibration amplitude *u* of the atoms decreases, and from Equation (2), the lattice resistance *τ_p_* to dislocations increases. The magnitude of *τ_µ_* is expressed as [33]:

(3) $$\tau_{\mu }=\alpha \mu b\rho^{1/2}$$

where *α* is a constant, *ρ* is the dislocation density, *b* is the Burgers Vector, and *µ* is the shear modulus. When the process of dislocation annihilation to counteract dislocation storage is suppressed during low-temperature deformation, the amount of stored dislocation increases significantly, increasing dislocation density *ρ*. Therefore, from Equation (3), it is evident that the resistance *τ_µ_* generated by the dislocation stress field increases as the ambient temperature decreases. In summary, Equation (1) explains that as the ambient temperature drops, the resistance *τ* to dislocation sliding in the aluminum matrix increases, the critical value of the applied stress required for the dislocation to start sliding increases, and the alloy strength also increases. The stress concentration *τ’* generated by the dislocation pile-up group at barriers (fixed dislocations, second phases, grain boundaries, etc.) is expressed as [34]:

(4) $$\tau^{’}=n\tau_{0}$$

where *n* is the number of dislocations in the dislocation pile-up group and *τ_0_* is the value of the tangential stress in the slip direction. Therefore, at low temperatures, the stress concentration in the alloy increases with the sliding of dislocations. The alloy fractures before the partial dislocation near the tensile fracture can slip any further, demonstrating that at low temperatures, the number of slip bands near the tensile fracture of the alloy decreases. Furthermore, decreasing temperature contributes to increased work hardening. These are the probable reasons for the increase in strength and decrease in the ductility of the alloy at low temperatures.

## 4. Conclusions

(1) The addition of Er elements to the Al-10Si-0.3Mg alloy increased the tensile and yield strengths of the alloy to 212 MPa and 155 MPa, respectively, at −60 °C, and the elongation peak at 8.35%. Adding Cu elements increased the tensile and yield strengths of the alloy to 284 MPa and 252 MPa at −60 °C, but the elongation decreased to 2.2%. After adding Cu-Er composite elements, the tensile and yield strengths of the alloy showed peaks at 295 MPa and 264 MPa, respectively, at −60 °C, but the elongation of the alloy was slightly decreased to 3.98%.

(2) The effects of modification and grain refinement were observed when the rare earth Er was added to the Al-10Si-0.3Mg alloy. The addition of Er elements changed the Si phase in the alloy from coarser, long, rod-like, and plate-like grains to fine, short, rod-like grains, and the SDAS was reduced. After the addition of Cu elements, a long strip form with an irregular net-like and minutely granular Al_2_Cu phase, Al_1.9_Cu_1.0_Mg_4.1_Si_3.3_ quaternary phase, and Cu-rich phase appeared in the alloy, acting as the second phase strengthening. The addition of Cu-Er elements improved the Si phase morphology and refined the grains in the alloy. However, no significant changes were observed in the other second-phase morphology.

(3) The decrease in the temperature decreased the atomic thermal vibration amplitude. At the same time, the dislocation stress field resistance and the stress concentration generated by the dislocation pile-up group at the barrier increased. Therefore, compared to 20 °C, the alloy of the same composition exhibited increased strength and decreased elongation when stretched at −60 °C, reducing the number of slip bands near the fracture.

## Figures and Tables

**Table 1:** Chemical composition and grouping of Al-Si alloys (wt.%).

Grouping of Alloys	Alloys	Si	Mg	Cu	Er	Al
Alloy A	Al-10Si-0.3Mg	10.030	0.296			Bal.
Alloy B	Al-10Si-0.3Mg-0.2Er	9.980	0.302		0.214	Bal.
Alloy C	Al-10Si-3Cu-0.3Mg	10.070	0.310	3.070		Bal.
Alloy D	Al-10Si-3Cu-0.3Mg-0.2Er	10.112	0.306	3.212	0.237	Bal.

**Table 2:** Effect of alloying elements on secondary dendrite arm spacing (SDAS) in Al-Si alloys with different composition.

Alloys	SDAS (µm)
Al-10Si-0.3Mg	18.1
Al-10Si-0.3Mg-0.2Er	16.9
Al-10Si-3Cu-0.3Mg	18.8
Al-10Si-3Cu-0.3Mg-0.2Er	17.3

## Data Availability

Not applicable.
